# Anthelmintic resistance in sheep gastrointestinal nematodes in Slovakia detected by in-vitro methods

**DOI:** 10.1186/s12917-014-0233-4

**Published:** 2014-10-01

**Authors:** Michaela Dolinská, Oksana Ivanišinová, Alžbeta Königová, Marián Várady

**Affiliations:** Institute of Parasitology, Slovak Academy of Sciences, Hlinkova 3, Košice, 040 01 Slovak Republic; Institute of Animal Physiology, Slovak Academy of Sciences, Šoltésovej 4-6, Košice, 04001 Slovak Republic

**Keywords:** Anthelmintic resistance, Sheep nematodes, Egg hatch test, Larval development test

## Abstract

**Background:**

The intensive use of anthelmintics for the control of helminthic infections has resulted in the development of anthelmintic resistance, which has become a major practical problem in many countries. A variety of tests are available to monitor anthelmintic resistance but most of them are expensive, laborious and time consuming and therefore unpractical for large field surveys. The main aim of this survey was thus to detect the occurrence of benzimidazole (BZ) and macrocyclic lactone resistance on sheep farms in Slovakia by using novel and modified in vitro methods that are inexpensive, easy to use and quick and therefore practical for large surveys.

**Results:**

BZ-resistant gastrointestinal nematodes were found on all 27 farms. Two farms (7.4%) had high levels of resistance (>40% of hatching), and 22 farms had low levels (<20% of hatching) of resistant nematodes. IVM-resistant populations were found on 14 of 49 sheep farms. The prevalence of BZ and IVM resistance has slightly increased on Slovak sheep farms during the last two decades.

**Conclusions:**

Both the BZ and IVM surveys indicated that resistance against anthelmintics was present on Slovak sheep farms. Resistance against the BZ class of anthelmintics had been stable for two decades, but a slight increase on IVM resistance was confirmed. Farmers must thus observe the preventive measures to avoid a faster onset of IVM resistance, otherwise the presence of resistant parasites and ineffective treatment may harm the economy of their farms.

## Background

Resistance to anthelmintic compounds in gastrointestinal parasites of sheep is presently cosmopolitan, and single- and multiple-drug resistant species are common throughout the world [1]. The reports of anthelmintic resistance (AR) in European countries mainly concern resistance against benzimidazoles (BZs), with local reports of levamisole resistance and isolated cases of resistance to macrocyclic lactones (MLs) [2,3]. BZ resistance has notably not yet reached a critical level in some European countries, e.g. Greece [4], Spain [5], Sweden [6], Italy [7] and Slovakia [8]. The extent and significance of AR in sheep in Slovakia have been previously studied in two national surveys. BZ resistance in Slovakia was first investigated in a survey in 1991-1993 [9]. This survey examined 77 farms and documented the presence of BZ resistance on six (7.8%) of the farms by using an in vitro egg hatch test (EHT). A survey in 2003-2004 [8,10] identified resistance to BZs on two of 29 farms (6.9%) by using a feacal egg count reduction (FECR) test, an in vitro EHT and a larval development test. Assuming that a low prevalence of BZ resistance persisted continuously between these two surveys, the status of BZ resistance appears not to have changed on Slovak sheep farms during that decade. The current extent of ivermectin (IVM) resistance in sheep parasites in the Slovak Republic is not known. Only one survey, in 2003-2004, covering 26 sheep farms has been conducted [8]. Resistance to IVM was suspected on eight of these farms (30.8%).

The growing importance of AR has led to an increased need for reliable and standardized methods of detection [11-13]. Effective monitoring of resistance is vital for maintaining a high efficacy of the currently available anthelmintics and for preventing further selection for resistance, especially in areas where AR is present in only a small proportion of the worm population. The use of a discrimination/delineating dose (DD) in the EHT provides a good estimate of genotypic resistance [14]. The egg hatch discrimination dose test (EHDDT) is less time-consuming, allows the reliable detection of a frequency of resistance alleles below 10% and is fairly reliable for the detection of BZ resistance under field conditions [14]. The micro-agar larval development test (MALDT) for detecting IVM resistance provides comparable and reliable results and can detect low proportions of resistant worms in populations, a sensitivity that should have potential in determining resistance with field tests [15,16].

The most commonly used drugs in Slovakia have been BZ and ML anthelmintics [17]. The main aim of this survey was thus to detect the occurrence of BZ and ML resistance on sheep farms in Slovakia by using novel and modified in vitro methods that are inexpensive, easy to use and quick and therefore practical for large surveys.

## Methods

### Trial design

Animal use and study design were approved by the Ethics Committee of the Institute of Parasitology of the Slovak Academy of Sciences in accordance with the national legislation in Slovakia - Animal Welfare Act No. 23/2009. Forty-nine sheep farms were investigated in 17 districts in Slovakia. Permission to collect study samples was granted by all participating sheep farms. The sheep flocks consisted of 300-800 animals on each farm. The farms mainly kept Tsigaja, Improved Valachian and Merino breeds. The animals examined were lambs or yearlings that had not received any anthelmintic treatment for at least 10 weeks before the initiation of the tests. On each farm, 15-20 animals were selected, and fecal samples were collected directly from the rectum. Pooled fecal samples weighing 50-100 g were stored anaerobically at room temperature [18] and processed within a maximum of two days. Nematode eggs were collected by sequentially sieving the faeces through three stacked sieves with decreasing apertures of 250, 100 and 20 μm. The material collected on the 20-μm sieve was washed with tap water and sedimented, after which the trichostrongylid eggs were recovered by the salt flotation method [19] and used for in vitro EHDDTs and MALDTs.

### Egg hatch discrimination dose test

The EHDDT was performed as described by Coles et al. [20]. A stock solution of thiabendazole (TBZ) (Sigma-Aldrich, Germany) was prepared by dissolving the pure compound in dimethyl sulfoxide (DMSO). The final concentration was prepared by adding 10 μl of the TBZ solution to 1.99 ml of an aqueous suspension of approximately 150 eggs∙ml^-1^. A single TBZ concentration of 0.1 μg∙ml^-1^ was used. A control (0.5% DMSO) without anthelmintic was also included in the test. The dosed egg suspensions were incubated in 24-well plates (Nuncleon, Denmark) for 48 h at 27°C. The incubation was then terminated by adding 10 μl of Lugol’s iodine to each well, and the numbers of eggs and larvae were counted. The test was performed with two replicates.

### Micro-agar larval development test

We used the method described by Coles et al. [13]. Tests were performed in 96-well microtiter plates. As we demonstrated in our previous study, the use of avermectin analogs and especially IVM aglycone significantly increased the ability of the test to differentiate between IVM-resistant and -susceptible isolates [15,16]. Stock drug solutions of IVM aglycone (Tebu-bio, France) with concentration of 13,02 μg. ml^-1^ were serially diluted 1:2 with DMSO to produce 12 final concentrations ranging from 0.084 to 173.6 ng∙ml^-1^. Subsequently, 12 μl of the stock solution were mixed with 150 μl of 2% Bacto agar. After solidification of the agar, 10 μl of eggs in a 0.3 mg∙ml^-1^ solution of amphotericin B (final number of eggs per well was 50-80) were mixed with 10 μl of yeast extract and then added to the agar. Only DMSO (1.3%) was used in the control wells. Yeast extract was prepared as described by Hubert and Kerboeuf [21] (1 g of yeast extract in 90 ml of 0.85% NaCl was autoclaved for 20 min, and then 27 ml of this solution were mixed with 3 ml of 10× concentrated Earle's solution). The plates were incubated for seven days at 25°C. Larvae were then killed with Lugol's iodine solution, and the number of third-stage (L3) larvae at each concentration was determined under an inverted stereomicroscope. The L3 larvae were morphologically differentiated and identified as by Van Wyk et al. [22].

## Results

### Egg hatch discrimination dose test

We examined 27 sheep farms in 2011-2012 in six districts of Slovakia for BZ resistance in gastrointestinal nematodes. Table 1 shows the percentages of hatched eggs for the farms obtained by the EHDDT. Instead of using the conventional threshold values (ED_50_ or ED_99_), we used the number of hatched eggs at a DD concentration of 0.1 ug∙ml^-1^ TBZ, because the results of Coles et al. [13] and Čudeková et al. [14] suggested that this DD prevented 99% of the susceptible eggs from hatching. The percentage of hatched eggs is thus a direct measure of the percentage of BZ-resistant eggs in the sample. The percentages were divided into four categories of farms based of their status of susceptibility/resistance determined by hatching in the EHDDT. More than 10% of the eggs from 12 farms hatched, and 10-20% of the eggs from 10 farms hatched. The hatching percentages for the DD on three farms were 20-40%. High hatching percentages (>40%) were recorded on only two farms.

**Table 1:** **Number of sheep farms with different percentage levels of hatched eggs at a threshold DD of 0.1 ug**∙**ml** ^**-1**^ **TBZ in EHDDTs on 27 farms**

	**Percentage of hatched eggs at a DD of 0.1 ug.ml** ^**-1**^ **TBZ**			
	(<10%)	(10-20%)	(20-40%)	(>40%)
# of farms	12	10	3	2

### Micro-agar larval development test

The results of the MALDTs from 49 farms are presented in Table 2. We chose the threshold discriminating concentration of 21.6 ng∙ml^-1^ IVM aglycone because no development of the susceptible isolates occurred at this concentration [15,16]. L3 larvae from 14 farms (28.57%) developed in this threshold concentration, which suggested the presence of IVM-resistant nematodes. A low level of resistant parasites (<30% development) was detected on 12 farms (24.49%). The presence of more than 30% resistant parasites was confirmed on only two farms (4.08%). The differentiation of L3 larvae at the discrimination concentrations revealed the presence of *Ostertagia*/*Trichostrongylus* spp. in all 14 tests. The MALDT was negative from 32 farms. Data could not be obtained from three farms due to low egg counts.

**Table 2:** **Number of sheep farms with the development of larvae at a threshold DD of 21.6 ng**∙**ml** ^**-1**^ **IVM aglycone in LDTs on 49 farms in Slovakia**

**# of sheep farms**	**Low egg counts in fecal samples**	**No resistance**	**Low resistance (<30% development)**	**High resistance (>30% development)**
49	3	32	12	2

## Discussion

The first survey of BZ resistance on Slovak sheep farms was conducted in 1991-1993 [9]. This survey examined 77 farms and documented the presence of BZ resistance on six farms (7.8%) by the use of the EHT. A further survey was performed in 2003-2004 [8,10] using in vivo and in vitro tests and found resistance to BZ products on two of 29 farms (6.9%). The status of BZ resistance on Slovak sheep farms changed little between 1993 and 2006, assuming a low persistent prevalence of BZ resistance during the intervening, untested period.

Our survey of BZ resistance was performed in 2011-2013, when we examined 27 sheep farms in six districts of Slovakia. We used the EHDDT to determine the percentage of resistant larvae in the populations by using a DD of TBZ (0.1 μg∙ml^-1^). We found resistant nematodes on all farms. Three separate field surveys of AR in parasites of sheep farms over almost two decades suggested that the percentage of farms with resistant nematodes was stable below 10%. All three surveys had used different classifications of BZ resistance. The farms in the first survey [9] used the WAAVP classification [20] that defined resistance as ED_50_ values in the EHT over 0.1 μg∙ml^-1^ TBZ. The second survey [8,10] defined resistance as in vivo FECRs less than 95% and lower 95% confidence limits of reduction less than 90%. Additionally, in vitro EHTs and LDTs were used in the survey, and farm populations were classified as resistant if the estimated ED_50_ was greater than 0.1 μg∙ml^-1^ TBZ in the EHT and if LC_99_ was over 0.03 μg∙ml^-1^ TBZ in the LDT. The present survey confirmed the presence of BZ resistant nematodes on all farms. Controversially, the results obtained in 1993 and 2012 are not necessarily different. The majority of the farms in 2013 had hatching percentages below 40%, which estimated ED_50_ to be below 0.1 μg∙ml^-1^ TBZ. Only two of 27 farms (7.4%) would have exceeded the threshold of 0.1 μg∙ml^-1^ TBZ of the second survey. Based on the questionnaire study of Slovak sheep farms [17], BZs are still in common use, due to the lower price compared to ML drugs. BZs had been widely used on sheep farms in recent decades, which undoubtedly contributed to the development of BZ resistance.

The first report of IVM resistance in Slovakia found resistant nematodes on six of 26 sheep farms (23.08%) and suspected resistance on eight more farms using in vivo FECRTs [8]. Our survey found a high occurrence (>30% resistant larvae using a DD) of IVM-resistant parasites on two farms (4.35%) and the presence of low resistance (<30% resistant larvae using a DD) on 12 farms (26.07%). IVM resistance was not detected on 32 of the 46 farms. Čerňanská et al. [8] concluded that the main reason for the unexpectedly high occurrence of IVM resistance on Slovak sheep farms could be due to a lower efficacy of the generic IVM formulations used in the survey. Similarly, van Wyk et al. [23] identified a problem of substandard generic products in South Africa. The occurrence of IVM resistance may thus be lower, as the authors described [8]. BZs and MLs are the most commonly used drugs in Slovakia for drenching sheep. The frequent use of generic IVM formulations in Slovakia during the last two decades, due to their low cost, may result in the lower efficacy. A direct comparison between our results and those of previous surveys is not possible due to the different methodologies used. The correlation between in vivo and in vitro tests is not always good. Maharshi et al. [24] observed a poor correlation between FECRs and the EHT for detecting BZ resistance. Phenotypic expression in the EHT was evident, with an occurrence of >30% homozygous resistant genotypes in the population. Based on our previous studies [25,14,16], the two in vitro tests, EHT and LDT, have the potential to detect a low level of resistance by using the ED_99_/LC_99_ criterion or a DD. The ED_50_/LC_50_ criterion, however, is not able to provide early detection during the development of resistance, especially if the alleles for resistance are rare in the parasite population. Additionally, the in vivo FECR test lacks the sensitivity to detect low levels of resistance [26,27].

The two subclasses of MLs, the avermectins and the milbemycins, are the most commonly used drugs against gastrointestinal parasites in small ruminants in most countries. These drugs entered the anthelmintic market in the 1980s, and IVM-resistant strains of gastrointestinal parasites of sheep have since emerged in many parts of the world. The situation in other European countries is similar to that in Slovakia, where the problem of IVM resistance has not yet reached a critical level. Documented cases of IVM resistance demonstrate that the situation is similar to the status in countries such Australia, New Zealand and South Africa 10-15 years ago. These countries are currently fighting the extreme spread of IVM-resistant parasites on sheep farms, but the reliance on a new anthelmintic class (e.g. amino-acetonitrile derivatives) is not always a solution [28]. All available measures must be used to prevent a repetition of history and to guard against the spread of IVM-resistant strains on sheep farms.

Diagnostic tests should be simple and inexpensive, provide rapid and reliable results and be suitable for standardization among the laboratories of many countries [16]. Both versions of the in vitro tests we applied for surveying field resistance provided easy, sensitive, rapid, inexpensive and repeatable diagnostic tools for diagnosing BZ and IVM resistance in the gastrointestinal parasites of sheep. The discriminating doses we used in our study offer valuable information for reducing the effort needed to determine the presence of resistance in parasite populations.

## Conclusions

Both the BZ and IVM surveys indicated that resistance against BZ and ML anthelmintics was present on Slovak sheep farms. Resistance against the BZ class of anthelmintics had been stable for two decades, but a slight increase on IVM resistance was confirmed. Farmers must thus observe the preventive measures to avoid a faster onset of IVM resistance, otherwise the presence of resistant parasites and ineffective treatment may harm the economy of their farms.
